# Amorphous solid dispersion formation via solvent granulation – A case study with ritonavir and lopinavir

**DOI:** 10.1016/j.ijpx.2019.100035

**Published:** 2019-11-12

**Authors:** Niraj S. Trasi, Sonal Bhujbal, Qi Tony Zhou, Lynne S. Taylor

**Affiliations:** Department of Industrial and Physical Pharmacy, College of Pharmacy, Purdue University, West Lafayette, Indiana 47907, United States

**Keywords:** Amorphous, Ritonavir, Lopinavir, Solvent granulation

## Abstract

Herein, we evaluate the potential of using a simple solvent granulation process to prepare a binary drug amorphous solid dispersion (ASD) containing two anti-HIV drugs, ritonavir and lopinavir. The drugs were granulated onto a mixture of lactose and microcrystalline cellulose, followed by drying to remove the solvent. The resultant granules were characterized and each drug was found to be X-ray amorphous. No crystallization was observed following storage for 1 month under accelerated stability conditions (40 °C and 75% relative humidity). The dissolution behavior of the compacted granules was compared with the marketed formulation. The dissolution rate of ritonavir was found to be significantly retarded relative to the commercial product when the two drugs were co-granulated. However, comparable release could be achieved when each drug was individually granulated, followed by combination and compaction. The solvent granulation approach may be a viable method to make ASDs of low dose drugs with low crystallization tendencies.

## Graphical abstract

**Figure:**
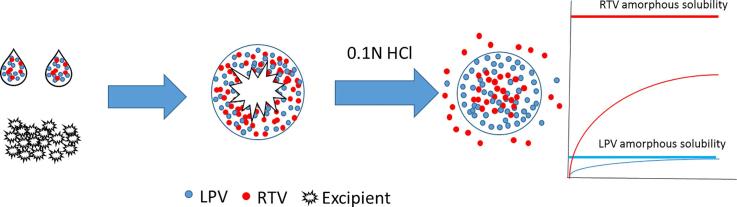

## 1 Introduction

With an increase in the number of therapeutically active compounds exhibiting low water solubility, formulation strategies such as amorphization, salt formation, complexation, micellization, are increasingly employed to improve solubility and bioavailability (Aisha et al., 2012, Frank et al., 2012a, Loftsson, 2017, Takano et al., 2010). Among these, amorphous formulations, which typically yield a higher solubility (supersaturated solution) and dissolution rate as compared to the crystalline state are often a preferred strategy (Hancock and Zografi, 1997). Other approaches such as complexation or micellar solubilization can increase the drug concentration in solution but do not necessarily result in supersaturation and thus will not improve the flux across a biological membrane (Frank et al., 2012b, Raina et al., 2015); flux has been shown to depend on the supersaturation gradient across the membrane rather than the concentration difference (Borbás et al., 2016). Since amorphous drugs tend to transform to the more stable crystalline phase upon storage, appropriate polymers are usually added to form a molecular dispersion, thereby decreasing the molecular mobility of the system, increasing the glass transition temperature (Tg), and providing kinetic stabilization against crystallization (Kothari et al., 2015, Prasad et al., 2014).

While there have been many investigations of amorphous solid dispersions (ASD) containing a single drug, an emerging scenario is the formulation of co-amorphous dosage forms. For these systems, there is a need to understand the impact of having more than one drug in the formulation. Multidrug formulations are becoming increasingly prevalent for diseases such as human immunodeficiency and hepatitis C viral infections, whereby treatment regimens require the administration of multiple drugs. These formulations either take advantage of a synergistic effect of the two drugs (Zhang et al., 2000) or improve the absorption of one drug by including another compound that interacts with and inhibits the efflux transporters or to inhibit metabolic enzymes (Drewe et al., 1999). Even though a multiple drug regimen can be achieved by simultaneous administration of separately formulated drugs, it may be preferable to co-formulate two or more drugs as fixed dose combinations (FDC) to improve patient compliance. A number of such FDC dosage forms are marketed including Kaletra® (lopinavir/ritonavir), Atripla® (efavirenz/emtricitabine/tenofovir disoproxil fumarate) and Harvoni® (sofosbuvir/ledipasvir). Some of these FDC products contain drugs that are crystalline in nature (e.g. Coartem®) while others have drugs that are formulated as amorphous solids to take advantage of their improved bioavailability, for example lopinavir and ritonavir. Recently, attention has been directed towards drug-drug co-amorphous systems wherein many combinations have been found to give a dissolution and physical stability advantage over the individually formulated compounds. One example is naproxen which, though generally resistant to amorphization, could be made amorphous by mixing with indomethacin (a drug in the same therapeutic class) (Löbmann et al., 2011). Improved stability of the co-amorphous system can be due to the formation of favorable intermolecular interactions between the two drugs thereby resulting in a higher than expected T_g_ (Martinez et al., 2016). Alternatively, it may be due to interference of the nucleation process as inferred in the case of nimodipine:nifedipine co-amorphous blends wherein the T_g_ of the mixture was lower than expected but the system was nevertheless more resistant to crystallization (Knapik-Kowalczuk et al., 2018). However, in terms of dissolution assessment, in many cases, comparisons are made between the co-amorphous system and the corresponding crystalline forms (Dengale et al., 2014, Shayanfar and Jouyban, 2013, Tantishaiyakul et al., 2009). Occasions wherein the dissolution performance is improved relative to that of the pure amorphous drug are usually seen in cases where the amorphous co-former is acidic and the drug is a base, hence salt formation occurs, or the acidic component increases the solubility of the drug by modifying the micro-environmental pH of the solute–solvent interface (Fung et al., 2018). In other instances, the individual amorphous drug crystallized faster when compared to the co-amorphous mixture and thus the dissolution advantage can be attributed to kinetic factors (Allesø et al., 2009). However, it has been shown previously that, when two drugs are miscible and uncharged, the amorphous solubility of each component in the mixture is invariably lower than that of the pure individual amorphous components due to a decrease in their chemical potential resulting from molecular level mixing (Alhalaweh et al., 2016; Trasi and Taylor, 2015a, Trasi and Taylor, 2015b).

Amorphous intermediates are usually manufactured at commercial scale using one of two main methods namely solvent evaporation (typically spray drying) or hot melt extrusion (HME) (Paudel et al., 2013, Repka et al., 2008) whereby each process has both advantages and limitations. HME requires that the drug is chemically stable at the high processing temperature employed (typically greater than 120 °C) and possesses a melt temperature that allows processing with a stabilizing polymer. Spray drying requires organic solvents to dissolve both the drug and the polymer leading to issues with controlling residual solvent content, and high costs associated with solvent recycling. Another drawback of spray drying is that the resultant powder may have a low bulk density and can have high electrostatic charge which can make powder handling difficult (Murtomaa et al., 2004). Both of these approaches are cost intensive in the manufacturing sense with high capital equipment costs. Since many drugs for the treatment of infectious diseases are used extensively in low-income countries, there is a need to develop more economical formulations and manufacturing approaches that can be performed in the developing countries.

**Fig. 1:**
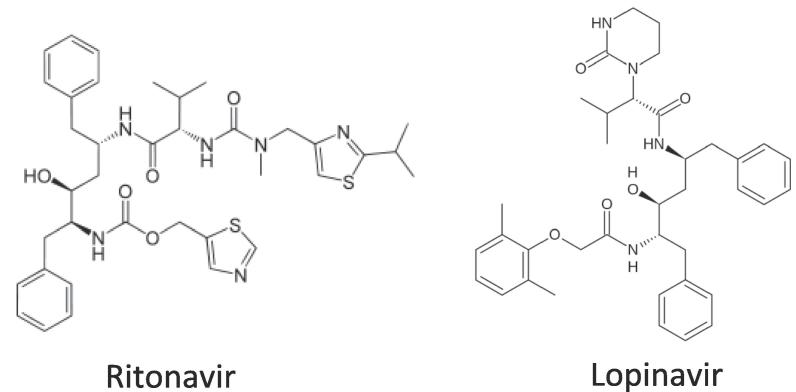
Molecular structures of ritonavir and lopinavir.

In this study, we have evaluated the possibility of developing an inexpensive manufacturing method to make amorphous solid dispersion formulations, wherein we studied the feasibility of formulating a fixed dose combination of lopinavir and ritonavir (Fig. 1) prepared using wet granulation/solvent impregnation. Various techniques including X-ray powder diffraction, infrared spectroscopy and release testing were used to characterize the resultant formulations.

## 2 Materials

Lopinavir and ritonavir were obtained from ChemShuttle (Hayward, CA). Microcrystalline cellulose pH 101 (MCC) and anhydrous lactose were obtained from Sigma-Aldrich (St. Louis, MO). Dichloromethane (DCM) and methanol (MeOH) were obtained from Fisher Scientific (Pittsburgh, PA). Cros-carmellose sodium was sourced from FMC Biopolymer (Newark, DE). Aluvia^TM^ (Manufactured by Abbott GmbH & Co, Ludwigshafen, Germany) was obtained from an Indian pharmaceutical distributor.

## 3 Methods

### 3.1 Preparation of dispersions

*Small scale experiments*. An excipient mixture consisting of a 1:1 wt blend of anhydrous lactose and MCC pH 101 was prepared by physical mixing using a vortex mixer. A 1:1 DCM:MeOH solution containing one or both of the drugs was then added to this mixture and granules were formed by mixing using a spatula. The two drug components were initially used at the ratio present in the marketed formulations, i.e. 200 mg LPV:50 mg RTV. Other ratios of the drugs were also subsequently studied to improve mechanistic understanding. 200 mg of LPV, 50 mg RTV, and 250 mg of PVPVA were dissolved in 2–3 mL of 1:1 DCM:MeOH and added to 1.5 g of 1:1 MCC:anhydrous lactose and mixed using a spatula. Formulations containing 5% w/w Span 20 (which is also present in the marketed formulation), or 0.1% w/w Tween 80 were also prepared whereby the overall tablet weight was maintained at 2 g. The granules were dried overnight in a vacuum oven, followed by milling for 10 s in a cryogenic mill (6750 freezer mill, Spex Sampleprep, Metuchen, NJ) to form a powder. RTV and LPV wet granulated dispersions were also prepared by making granules containing just one drug (1:1 drug:polymer ratio) using the following formula, 50 mg RTV + 50 mg PVPVA + 400 mg excipients and 200 mg LPV + 200 mg PVPVA + 1000 mg excipients. Preparation of the 20:80 drug:PVPVA dispersion was performed using a larger amount of excipient (3 g per tablet) as the base powder resulting in a total tablet weight of above 4 g. A summary of all formulations is provided in Table S1.

The resultant ASD formulation was placed in a desiccator with a saturated solution of sodium chloride which provides a relative humidity of 75%. The container was placed at 40 °C and samples were removed every 7 days and analyzed by XRPD to monitor crystallization.

*Bench-top granulation*. Bench-top granulation experiments were also performed to verify that granules could be produced using more industrial-like processing equipment. A custom-made bench-top granulator, described in detail previously, was used (Wikstrom et al., 2008). An excipient mixture consisting of 1:1 by weight blend of anhydrous lactose and MCC pH 101 was prepared using a V – blender. Sixty grams of this blend was placed in the granulator. A 1:1 DCM:MeOH solution containing both of the drugs (12 g LPV and 3 g RTV) and PVPVA (15 g) was then gradually added to the excipient blend using a peristaltic pump. The rate of solvent addition was 25 mL/min, and addition occurred in <4 min. The tip speed used was 94 m/sec. The solution was added until complete granulation of the powder occurred. The granules were sieved and dried overnight in a vacuum oven. The granules were subsequently assayed for their drug content confirming that a 10% drug loading was achieved. The granules were then characterized in terms of their Carr’s Index, Hausner ratio, angle of repose, and particle size (using sieve analysis). Tablets containing 150 mg of granules were prepared using a computer-controlled tablet press (Gamlen Tableting Ltd., Nottingham, UK) by direct compression. The granules were compacted with a target load of 500 kg at the speed of 120 mm/min using a 6 mm punch. The force-displacement data obtained during tablet preparation was analyzed to obtain the maximum load applied to the tablet during the compression, detachment and ejection steps. The tablets were evaluated for their hardness (Vanderkamp-Benchsaver Series, Cary, NC) and friability (Vankel Friabilator, Cary, NC). Both the tests were performed as per the specifications in USP.

### 3.2 X-ray powder diffraction (XRPD)

The X-ray diffraction profiles of the powders were determined using a Rigaku Smartlab^TM^ diffractometer (Rigaku Americas, Texas, USA) with a Cu-Kα radiation source and a D/tex ultra detector. Samples were loaded onto glass sample holders and powder patterns were obtained from 5 to 35° 2θ at a scan speed of 10°/min and a step size of 0.02°. The voltage and current used were 40 kV and 44 mA respectively.

### 3.3 Dissolution testing

The dispersions prepared at small-scale containing 200 mg of LPV and 50 mg of RTV were weighed and made into a compact using an E-Z hydraulic press (International Crystal Laboratories, Garfield, NJ) at 500 psi for 3 s before removing the pressure. Croscarmellose sodium (10 wt%) was added to ensure tablet disintegration. Compacts were prepared to improve wetting during dissolution testing as the powder had a tendency to float, and to provide a better comparison with the marketed formulation. Tablets were added to 250 mL of pH 6.8 10 mM phosphate buffer pH 6.8, maintained at 37 °C in a jacketed beaker and stirred with a magnetic stirrer at 150 rpm. Samples were removed at regular time intervals, filtered through a 0.45um PTFE syringe filter (Tisch Scientific, North Bend, OH) and analyzed by high performance liquid chromatography (HPLC) as described below. The dissolution of Aluvia^TM^ in pH 6.8 media was difficult to determine due to significant difficulty in filtering the dissolution medium. This was due to the formation of a milky solution during dissolution indicating the formation of nanodroplets which clogged small pore size filters and passed through filters with a larger pore size. Dissolution was also carried out in 250 mL of 0.1 N HCl under the same conditions to mimic dissolution in gastric media. For this medium, Aluvia^TM^ tablet dissolution also could be determined. For the scaled-up granules, granules equivalent to 100 mg of drug content were added to a dissolution media consisting of 100 mL of 0.1 N HCl in a jacketed beaker maintained at 37 °C and stirred with a magnetic stirrer at 150 rpm. Samples were removed at regular time intervals and filtered through a 0.45 μm PTFE syringe filter (Tisch Scientific, North Bend, OH) and analyzed by HPLC.

A pH shift dissolution experiment was performed on the marketed product by adding one tablet to 200 mL of 0.1 N HCl stirred at 150 rpm and held at 37 °C. After 30 min, the solution was neutralized with 750 mg of NaOH (dissolved in 10 mL of water) and an additional 40 mL of 100 mM pH 6.8 buffer was added and the dissolution was continued.

The amorphous solubility of the each drug in 10 mM pH 6.8 phosphate buffer was determined by preparing a stock solution of 20 mg/mL of RTV or LPV in methanol and adding 100 µL of the solution to the buffer containing 10 µg/mL HPMC (to inhibit crystallization) with stirring at 37 °C. The solutions were then ultracentrifuged using an Optima L-100 XP ultracentrifuge (Beckman Coulter Inc., CA, USA) with a SW 41 Ti swinging bucket rotor attachment at 35,000 rpm at 37 °C. The supernatant containing the molecularly dissolved drug was then removed and analyzed using high performance liquid chromatography (see below). The resultant concentration was taken as the amorphous solubility.

### 3.4 High performance liquid chromatographic (HPLC) analysis

HPLC analysis was performed using an Agilent HPLC 1260 Infinity II system (Agilent Technologies, Santa Clara, CA) equipped with a diode array detector. The column used for analyzing the samples was a Sunfire^TM^ C-18 analytical column (3.0 mm × 150 mm, 3.5 µm, 100 Å) (Waters Corporation, Milford, MA). The mobile phase was an acetonitrile: water mixture at a ratio of 60:40 v/v and the flow rate was 0.5 mL/min. The analysis was conducted at room temperature and the detection wavelength was 215 nm. The retention times for RTV and LPV were 3.9 and 4.5 min respectively. The concentration of the drug was determined from the area under the curve of the drugs by comparing to a standard curve prepared from a stock solution of the drugs, both of which had R^2^ values of 0.999.

### 3.5 Fourier transform Infra-red (FTIR) analysis

FTIR spectra of melt quenched RTV and LPV were obtained using a Golden Gate ATR accessory (Specac, Fort Washington, PA) installed in a Vertex 70 model IR Spectrophotometer (Bruker Optics, Billerica, MA). A total of 64 scans were averaged in the spectral range of 400–4000 cm^−1^. The ATR unit, as well as the detector compartment, were kept continuously flushed with dry air. The spectra of the co-amorphous systems were determined after mixing and melting 200 mg LPV and 50 mg RTV into a pellet. This pellet was then exposed to the 0.1 N HCl dissolution media for 1 h and then dried. The surface of the pellet that was exposed to the acidic media was scraped and the powder analyzed to determine the change in drug ratio on the surface.

## 4 Results

### 4.1 XRPD analysis and stability testing

**Fig. 2:**
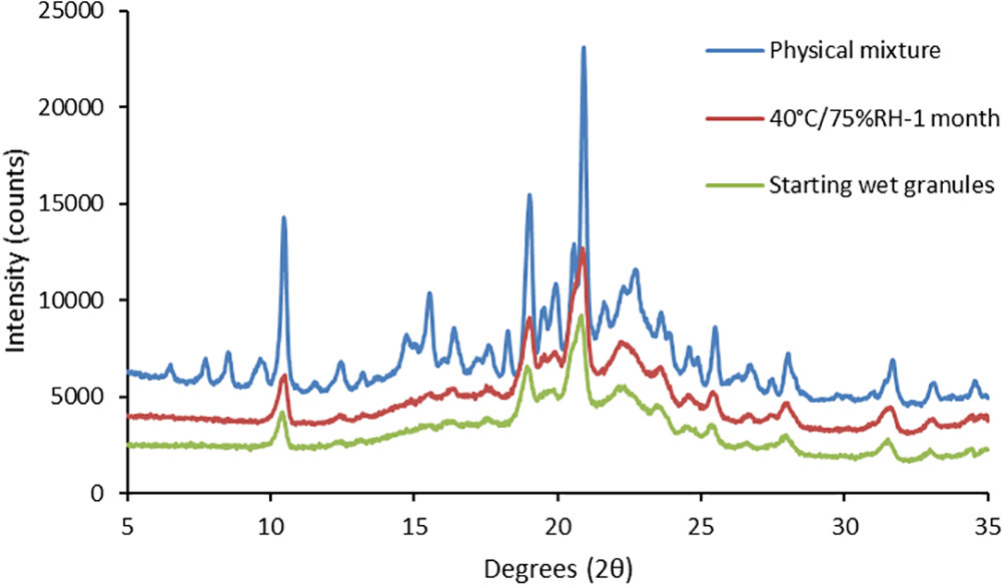
XRPD profiles of the granules before and after storage at accelerated storage conditions for 4 weeks shows that there is no development of crystalline peaks characteristic of either drug, whereby peaks arising from each drug can be clearly seen in the physical mixture.

X-ray diffractograms of the 200/50 LPV/RTV granules prepared with PVPVA showed an absence of crystalline peaks arising from either drug suggesting that the drugs were present in their amorphous form. Reference diffractograms of crystalline ritonavir, lopinavir, and a blend of lactose and MCC are shown in Figure S1. The Bragg peaks seen in Fig. 2, in the diffractograms of the granules, arise from lactose which is in its crystalline form in the granules. In contrast, a physical mixture of crystalline drugs and excipients at the same drug loadings yielded an XRPD pattern with clearly distinguishable drug crystalline peaks, for example there are several peaks appearing at values below 10° 2Θ that are due the presence of crystalline drugs; these are absent in the granules (Fig. 2). Storage of the granules under accelerated stability open dish conditions (40 °C/75%RH) for one month did not result in the appearance of any crystalline peaks indicating that the granules prepared by solvent evaporation were resistant to crystallization for this time period. When the drugs were granulated individually with a blend of MCC and lactose, no crystallization was observed after storage under similar conditions for one month (Figure S2).

### 4.2 Dissolution testing

#### 4.2.1 Dissolution in pH 6.8 buffer

**Fig. 3:**
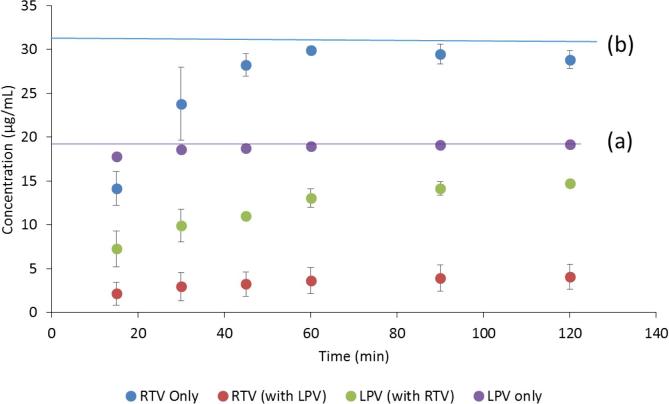
Dissolution profiles of ritonavir and lopinavir from granules when granulated individually and when co-granulated in pH 6.8 buffer. Horizontal lines indicates (a) lopinavir amorphous solubility and (b) ritonavir amorphous solubility.

The dissolution of RTV and LPV granules formulated as a single component and as a co-granulated formulation was evaluated in both near-neutral and acidic media, focusing on the maximum extent of supersaturation achieved. Under near-neutral pH conditions, the crystalline solubility of RTV is 1.8 µg/mL and that of LPV is 2.9 µg/mL, hence the dissolution conditions are highly non-sink with respect to the crystalline solubility. Neither compound is ionized at pH 6.8. Further, the dissolution data represent the concentration of molecularly dissolved species whereby the samples have been filtered to remove any colloidal aggregates. The resultant values can be used to calculate the extent of supersaturation, which is considered important for enhancing the rate of membrane flux *in vivo* (Bevernage et al., 2013). As can be seen from Fig. 3, RTV granules dissolved to a maximum concentration of just below 30 µg/mL, while LPV granules reached a final concentration of around 20 µg/mL at the end of 2 h of dissolution when the drugs were granulated and dissolved individually. These concentrations are close to the amorphous solubility of these compounds in this medium i.e. 31 µg/mL and 19 µg/mL for RTV and LPV respectively. Thus the solutions are maximally supersaturated with relative supersaturations (where relative supersaturation, S, is C/C* where C is the experimentally measured concentration and C* is the crystal solubility) of ~17 and ~7 for RTV and LPV respectively. However, when the 200/50 LPV/RTV granules containing a co-granulated intimate mixture of the drugs were dissolved, the maximum concentrations of each drug achieved were considerably lower, with the RTV concentration reaching a plateau at 4–5 µg/mL, while the LPV concentration reached around 15 µg/mL. Correspondingly, the supersaturation ratio was reduced to a factor of only ~3 for RTV, while that of LPV was reduced slightly to 5.

#### 4.2.2 Dissolution in 0.1 N HCl

**Fig. 4:**
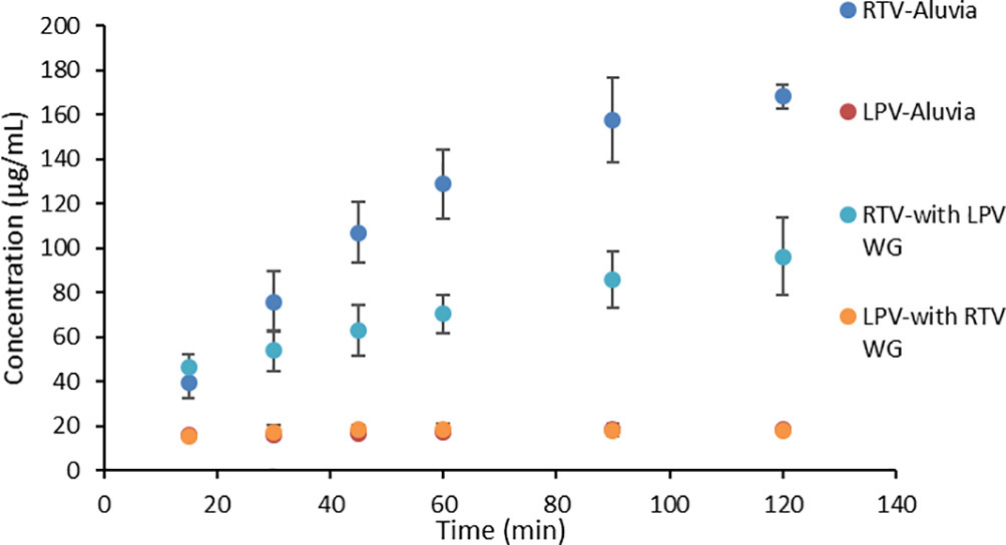
Dissolution of Aluvia tablets and co-granulated LPV-RTV 50:50 drug:polymer ASD compacts in 250 mL 0.1 N HCl at 37 °C.

RTV is a weakly basic drug with pKa values of 1.8 and 2.6 for the two thiazole moieties. Therefore, it is highly, although not completely, ionized at pH 1.2, resulting in increased solubility (Law et al., 2001). At pH 1.2 the solubility of crystalline RTV is ~0.4 mg/mL (Law et al., 2001) and for dissolution experiments at pH 1.2, sink conditions with respect to crystalline solubility exist. Consequently, when the dissolution is performed in an acidic media, significantly more RTV is molecularly dissolved relative to that in neutral media due to the higher solubility of the ionized form. The molecularly dissolved RTV concentration upon Aluvia^TM^ dissolution reached around 170 µg/mL at the end of 2 h while the LPV concentration remained close to its amorphous solubility (Fig. 4). LPV is un-ionized at this pH and therefore does not dissolve to a higher concentration relative to that observed in neutral media. Interestingly, despite the acidic dissolution medium providing sink conditions, the concentration of molecularly dissolved RTV at the end of the dissolution experiment is lower than expected indicating incomplete release; the expected final solution concentration if all of the RTV dissolved is 200 μg/mL. To determine the reason for this, a 200 µg/mL solution of RTV was prepared in 0.1 N by dissolving RTV-only granules and to this was added 0.8 mg/mL of LPV, prepared by dissolving LPV-only granules, and the system was stirred for one hour. The RTV free drug concentration after addition of LPV decreased by 10% to around 180 µg/mL, indicating that the presence of LPV induced a small amount of RTV precipitation (data not shown).

The release rate and extent of RTV from the tableted granules (where both RTV and LPV were co-granulated) was significantly lower than that from Aluvia^TM^ with the concentration being less than 100 µg/mL after 2 h, while the LPV release rate and extent were comparable (Fig. 3). The scaled-up co-granulated system likewise showed a lower release profile than observed from Aluvia^TM^ (Figure S3).

#### 4.2.3 Effect of pH shift on dissolution

**Fig. 5:**
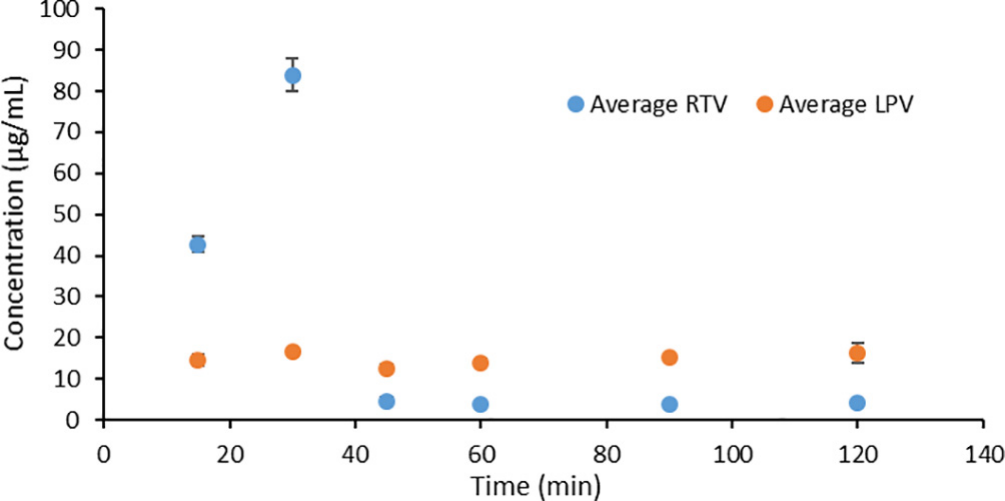
Concentration versus time profile following initial dissolution in acidic media (250 mL 0.1 N HCl) followed by an increase in solution pH to pH 6.8 after the 30 min time point. The RTV concentration is reduced due to a change in the ionization state of the drug, with precipitation, while the neutral LPV is only slightly impacted initially by the 25% dilution.

When the marketed formulation was first dissolved in acidic media and the pH subsequently increased, we observed a rapid decrease in the RTV concentration to below 5 µg/mL (Fig. 5), which cannot be accounted for by the modest dilution. The resultant level of supersaturation is similar to that observed for RTV released from the co-granulated ASD under neutral conditions (Fig. 3). At first glance, it might be assumed that this rapid decrease upon pH shift is due to the generation of an initially high supersaturation due to a decrease in the extent of ritonavir ionization, followed by rapid crystallization.

**Fig. 6:**
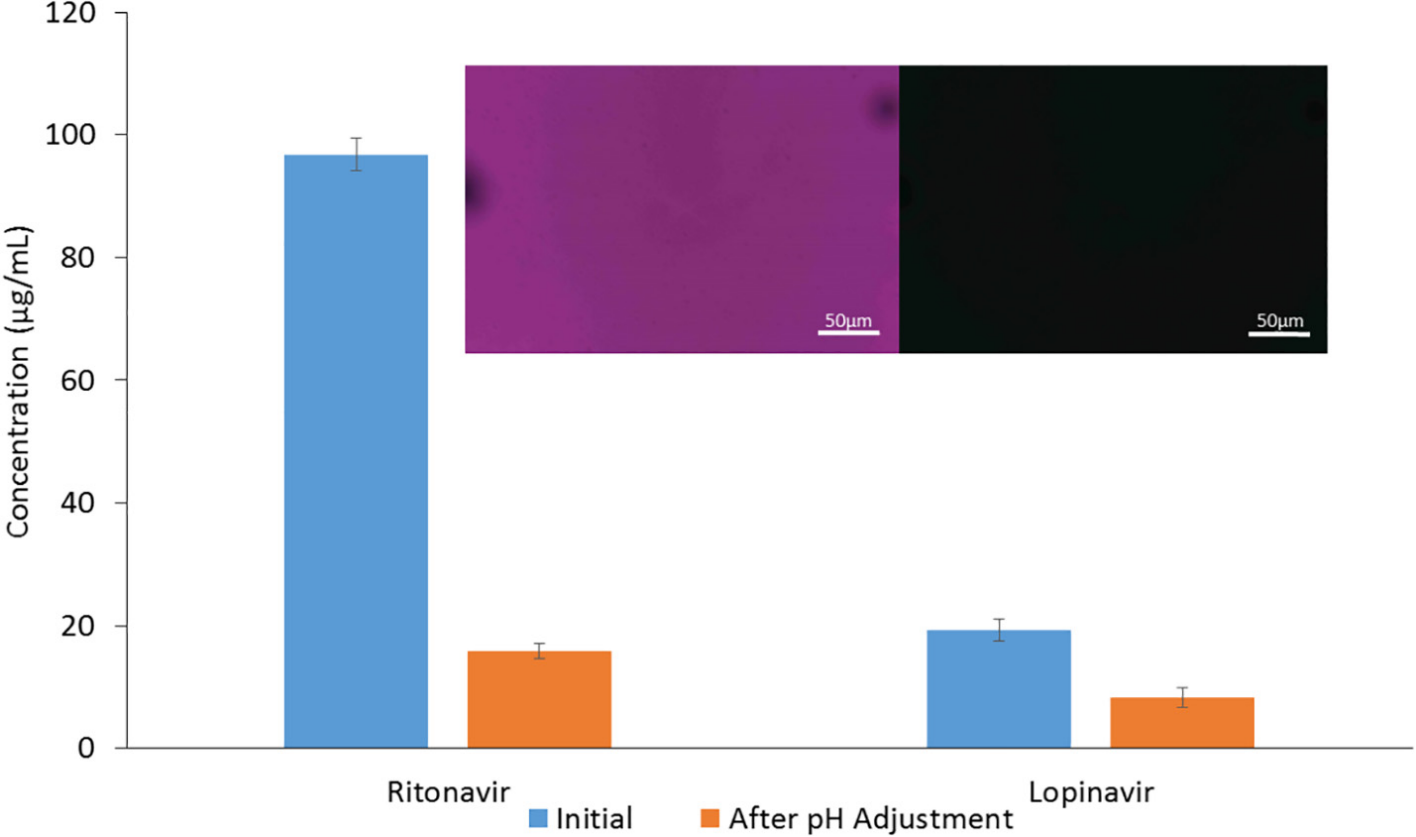
Change in the concentration of molecularly dissolved drug when the pH is shifted from 1.2 to 6.8. Concentrations were determined following ultracentrifugation to remove any precipitates. The inset shows the lack of birefringence of the observed drug precipitates indicating that precipitated material is non-crystalline. The dilution factor was 25%.

To better evaluate the crystallization tendency upon pH shift, this experiment was repeated in the absence of any excipients which can interfere with the ability to detect crystalline material, especially insoluble excipients such as MCC. Here, a solution containing 100 µg/mL RTV, and 400 µg/mL LPV, initially at pH 1.2 was neutralized by addition of NaOH to yield a final solution pH of 6.8. Following addition of NaOH, precipitation was observed. Some of the resultant suspension was ultracentrifuged, followed by analysis of the RTV and LPV concentrations in the supernatant. A portion of the remaining suspension was analyzed using a polarized light microscope to determine if crystals could be detected. The concentration of RTV in the supernatant dropped considerably following pH change, from 100 to less than 20 µg/mL (Fig. 6). The LPV concentration also decreased. However, no crystals were observed in the precipitated solution (Fig. 6 inset). These observations support the precipitation of a non-crystalline form of ritonavir following an increase in pH. However, this non-crystalline form has a lower solubility than amorphous RTV alone (which is around 32 µg/mL, Fig. 3), suggesting that LPV and RTV are mixed together in the precipitate when both compounds are unionized, which leads to a reduction in the maximum supersaturation, as observed previously (Trasi and Taylor, 2015a, Trasi and Taylor, 2015b). Further these observations are in general agreement with the granule dissolution experiments, both for the pH shift experiments shown in Fig. 5, and the single stage dissolution experiments on the co-granulated drugs shown in Fig. 3, whereby the RTV concentration at higher pH is considerably lower than the amorphous solubility of RTV alone.

#### 4.2.4 Effect of drug loading and surfactants on dissolution

Various strategies were employed to improve the dissolution profile of the wet granulated dispersions, so that a better match to the commercial product was achieved in terms of the molecularly dissolved drug concentrations in the stomach environment. First, the total drug loading (relative to polymer) was reduced from 50 wt% to 20 wt%. However, no overall improvement in release performance was achieved using this approach (data not shown). Next, addition of surfactants was evaluated; a surfactant is present in the commercial product and surfactants have been shown to improve release from amorphous formulations (Mosquera-Giraldo et al., 2018).

**Fig. 7:**
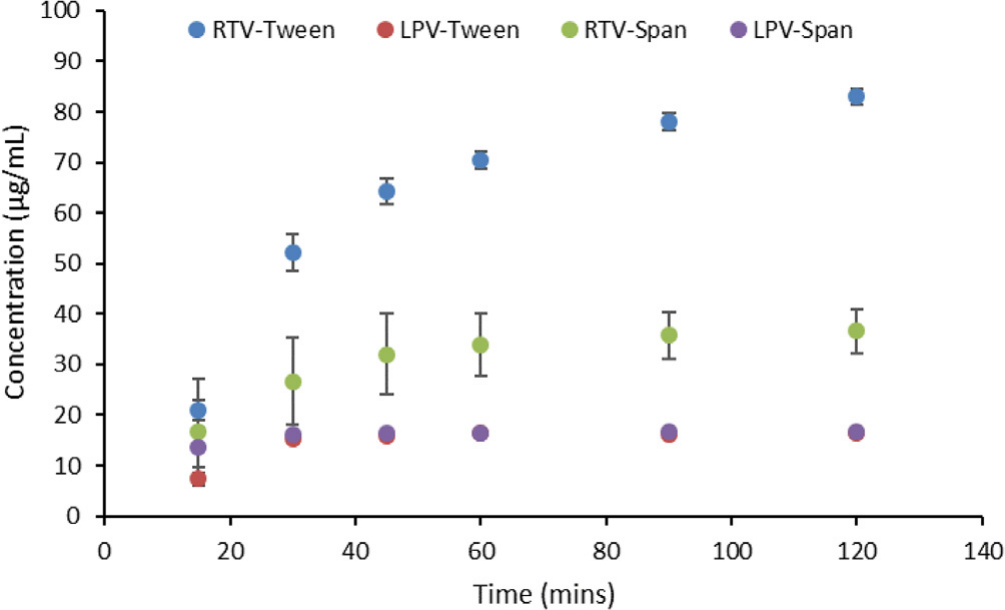
Dissolution of LPV-RTV granules at 1:1 drug:polymer ratio in 0.1 N HCl in the presence of 0.1% Tween and 5% Span 20 in the formulation.

As shown in Fig. 7, the addition of surfactants in the dispersions did not aid in improving the dissolution rate of RTV. The addition of Tween resulted in a similar profile to in the absence of the surfactant, while the addition of Span 20, present in the marketed formulation, seemed to reduce the rate and extent of RTV release. Neither surfactant had an appreciable impact on LPV release.

#### 4.2.5 Effect of LPV:RTV ratio on release

**Fig. 8:**
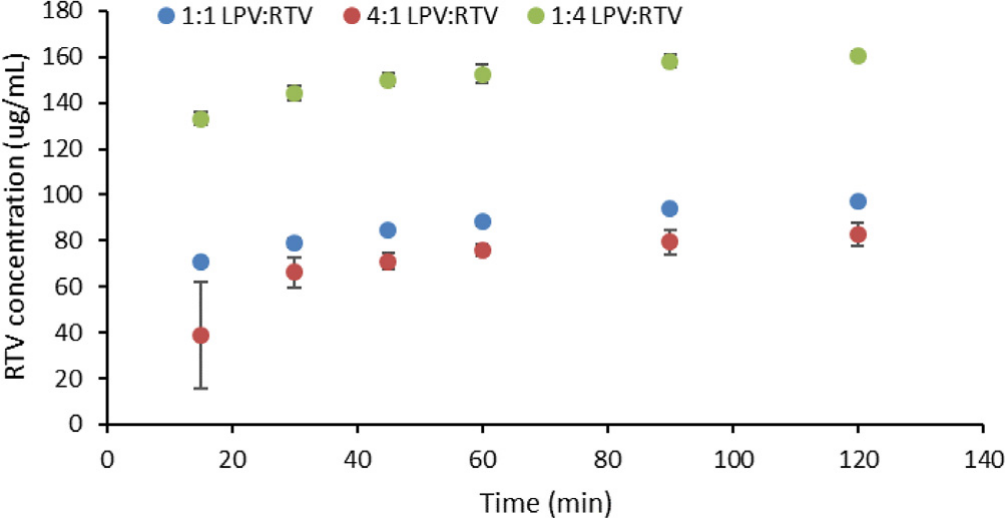
Dissolution of 50 mg RTV in 250 mL 0.1 N HCl for dispersions containing differing ratios of LPV and RTV.

To better understand the reduced solution concentrations of RTV in the presence of LPV (i.e. when both drugs are in the same ASD), we also prepared and evaluated granules with differing ratios of LPV and RTV, namely 125:125 and 50:200 LPV:RTV, in addition to the marketed 200:50 ratio. The dissolution of tablets made from these granules in 0.1 N HCl, such that the amount of RTV added to the media was the same in all the samples, showed that RTV reaches its highest concentration when it is prepared at the ratio 50:200 LPV:RTV (Fig. 8). Thus, for a constant total amount of RTV, a lower ratio of LPV enables enhanced RTV dissolution from the amorphous co-granulated mixture. This observation confirms that the presence of LPV, intimately mixed with RTV as in the co-granulated ASDs retards RTV release.

#### 4.2.6 Effect of granulating the drugs separately

**Fig. 9:**
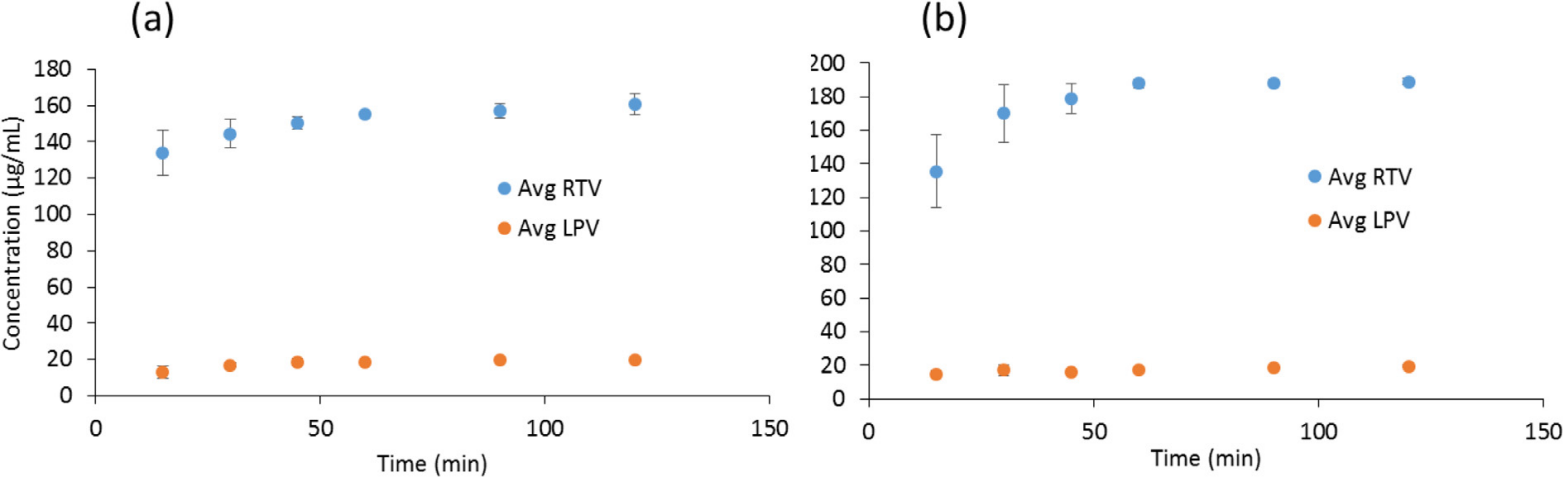
Dissolution from (200:50 LPV:RTV) tablets prepared by (a) mixing individually granulated RTV and LPV and then compressing into one tablet and (b) compressing the individually granulated powders separately as bi-layered tablets. Dissolution medium is 0.1 N HCl.

A different approach employed to prepare the FDC tablets in an effort to improve the release profiles was to use individually granulated powders. In other words, granules with only one drug in the dispersion were prepared and then blended and compacted. Alternatively, bi-layered tablets were prepared where the granules containing each drug were kept physically separated. As can be seen from Fig. 9, both of these approaches resulted in rapid RTV releases whereby the final concentrations achieved were similar to those seen for the Aluvia^TM^ formulation.

### 4.3 FTIR analysis to understand dissolution behavior

**Fig. 10:**
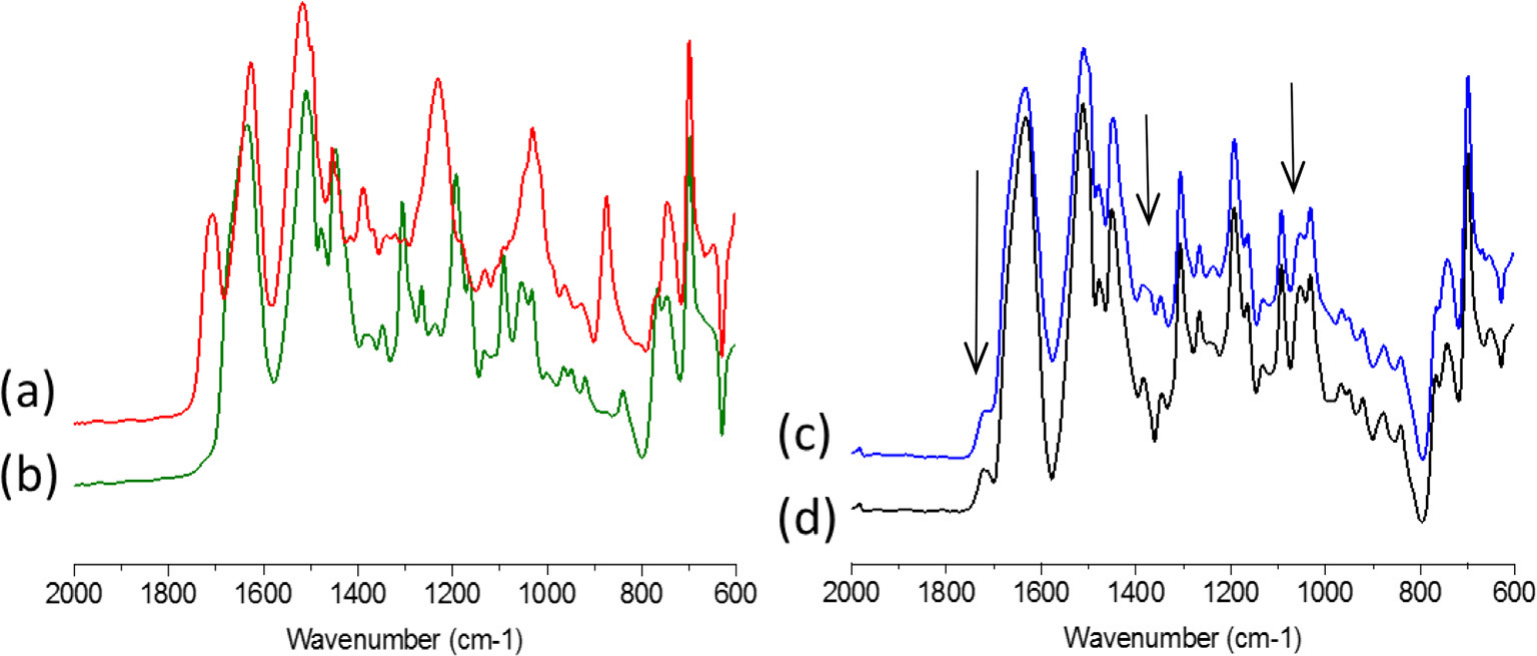
FTIR spectra of (a) pure amorphous RTV, (b) pure amorphous LPV, (c) 200:50 LPV:RTV melt quenched amorphous blend and (d) 200:50 LPV:RTV melt quenched amorphous blend after exposure to 0.1 N HCl for 1 h.

The FTIR spectra of the two drugs are very similar (Fig. 10) with the main differences observed in the carbonyl spectral region of 1575–1800 cm^−1^ where RTV has an additional peak at 1704 cm^−1^ adjacent to the overlapping peaks at around 1630 cm^−1^. RTV also has two large peaks at 1229 and 878 cm^−1^ which are absent in LPV. When the two drugs are mixed and melt quenched together at a ratio of 200:50 LPV:RTV, the peaks uniquely representative of RTV are hard to distinguish, presenting, for example, as a small shoulder at 1718 cm^−1^. Upon exposure to 0.1 N HCl and partial dissolution of the surface of the amorphous mixture, the intensity of the shoulder decreases and the ratio of peaks at 1630 cm^−1^ and 1718 cm^−1^ increases from 7.7 to 11.2, indicating a preferential loss of RTV from the surface during dissolution. In general, the spectrum becomes slightly more LPV-like after partial dissolution with additional subtle changes in the peaks, as shown by the arrows in Fig. 9. Given that the ATR sampling methodology is somewhat surface sensitive (Planinsek et al., 2006), these observations are consistent with surface enrichment of LPV due to preferential dissolution of RTV. This is also consistent based on mass balance considerations using the dissolution data under acidic conditions where it is apparent that relatively more RTV dissolves than LPV. Consequently, the amount of LPV in the undissolved ASD increases relative to the amount of RTV.

### 4.4 Properties of granules and tablets

The properties of granules produced using the bench-top granulator, and tablets prepared from these granules, were evaluated. The angle of repose value of granules was found to be 36.2 ± 0.7°. The Carr’s Index and Hausner ratio values were 14.4 ± 3.5% and 1.17 ± 0.05 respectively. Sieve analysis of granules indicated that more than 80% of the particles lie in the size range of 150–595 µm (Figure S4). The granules contained less than 1.5% of fines (particle size <45 µm). The calculated span value of granules was 1.72. The drug load % was found to be 8.4% for LPV and 2% for RTV. For tablet preparation, the maximum loads applied during compression, detachment and ejection steps were 518.7 ± 0.7 kg, 19.1 ± 3.1 kg, and 87.6 ± 4.4 kg respectively. All tablets were found to be intact after the friability test, with a friability of 0.7%. The hardness of the tablets was 10.17 ± 0.53 kPa. These results indicate that this manufacturing approach leads to granules with acceptable flow properties that can be compressed into tablets of low friability and sufficient hardness.

## 5 Discussion

Wet granulation using organic solvents appears to be a possible alternative approach to HME and spray drying for preparing an amorphous dosage form of the slowly crystallizing compounds, RTV and LPV, albeit with certain caveats. Thus, the amorphous forms of the drugs were successfully generated by solvent granulation/impregnation, and the resultant granules were found to be physically stable against crystallization. Moreover, granules with good flow and compression properties could be produced using this approach. However, there are factors that need to be considered when drugs are formulated and produced using this approach. One consideration is the final size of the tablet, since a relatively large excipient mass is required for successful granulation with the drug-polymer organic solvent solution to avoid formation of a sticky mass. Clearly important factors here include the dose of the drug, as well as the solubility of the drug and polymer in the solvent system employed. Thus, the wet granulation approach is likely to be best suited for low dose formulations, or those where the drug and polymer are highly soluble in the solvent system, allowing a lower volume of solvent to be used. This issue also can be potentially, at least partially, mitigated by using excipients with a higher solvent adsorption capacity; preliminary experiments showed that using a higher amount of MCC enabled an increase in drug loading. Alternatively, fluid-bed granulation is also a possible alternative, where a larger volume of solvent is less of an issue due to rapid solvent evaporation, facilitating a decrease in the amount of excipients. Of course, the drug also must be resistant to crystallization during the solvent evaporation process. Hence, more slowly crystallizing compounds, such as those with higher molecular weights and more complex structures, are most suitable for this general approach. Additionally, adequate drying is required to remove the organic solvent used to dissolve the hydrophobic drugs. The use of organic solvents to dissolve the drug and mix with the polymer in a simple manufacturing process is not without precedent. Tacrolimus amorphous solid dispersions (the commercial product is Prograf®) are prepared by dissolution of the drug in an organic solvent, solvent impregnation into the polymer, solvent removal, and then blending with additional excipients prior to filling into capsules (Honbo et al., 1987, Trasi et al., 2017, Yamashita et al., 2003). The wet granulation approach described herein is attractive since it can potentially reduce the number of required processing operations, producing granules that can be mixed with a disintegrant and then compressed.

In terms of dissolution performance, the co-granulated sample in the neutral media resulted in lower concentrations of both drugs relative to dissolution of single component granules (when dissolved alone), whereby the concentration of RTV was depressed more than that of LPV (Fig. 3). The reason for the lowered concentration upon dissolution of the co-granules is due to the mixing of the two compounds in the amorphous solid state, i.e. formation of a co-amorphous system. This results in a mutually decreased chemical potential, and thus a lowered amorphous solubility of each compound, which also decreases the driving force for dissolution, as described in detail previously (Trasi and Taylor, 2015b). In other words, mixing the drugs together to form a single phase amorphous system, is detrimental to the amorphous solubility of each drug. Further, RTV is affected to a greater extent than LPV. This is because the extent of the amorphous solubility reduction caused by mixing with a second compound in the amorphous phase is dependent on the relative proportions of each compound, with the minor component experiencing a greater extent of amorphous solubility reduction than the major component. Because RTV it is present in a lower amount in the co-granulate (the ratio of LPV:RTV is 4:1), it experiences a greater decrease in chemical potential, consistent with a greater extent of dilution in the co-amorphous system brought about by mixing with the larger amount of LPV. In other words, molecular mixing of both components into a single amorphous phase negatively impacts their dissolution performance (Alhalaweh et al., 2016a, Trasi and Taylor, 2015b), explaining the data presented in Fig. 3.

While the mutually reduced amorphous solubility explains the single step, pH 6.8 dissolution results (Fig. 3), the situation is much more complex for two-step dissolution, whereby the pH is initially acidic, and then increased to pH 6.8. This is because one of the components, RTV, can undergo ionization at low pH. Due to the higher solubility of the ionized form, RTV releases from the granules to a much higher concentration in acid relative to at neutral conditions. However, when the pH increases, RTV solubility decreases due to the change in ionization state, with the solution becoming supersaturated. Further, if the un-ionized concentration exceeds the amorphous solubility, as in this study, liquid liquid phase separation with the formation of amorphous nanodroplets occurs (Ilevbare and Taylor, 2013, Sugihara and Taylor, 2018). Given that both RTV and LPV are present in the solution phase following pH change, mixing of the neutral drug species occurs. Mixing between RTV and LPV in the nanodroplets will result in the same amorphous solubility (and hence concentration) suppression phenomenon described above. This can be clearly seen with the pH switching experiments shown in Fig. 5, Fig. 6, where only the molecularly dissolved drug was measured (the precipitated material was removed by centrifugation). Under acidic conditions, high RTV concentrations are achieved upon dissolution due to ionization, while after the pH increase, the molecularly dissolved RTV concentration (i.e. the extent of supersaturation) plummets. This decrease can be attributed to three factors, 1) a modest dilution factor (25%), 2) a change in ionization state which leads to the amorphous solubility being exceeded, and hence the formation of a drug-rich phase with the loss of free drug from solution and, 3) suppression of RTV amorphous solubility by mixing of RTV and LPV in the nanodroplets.

**Fig. 11:**
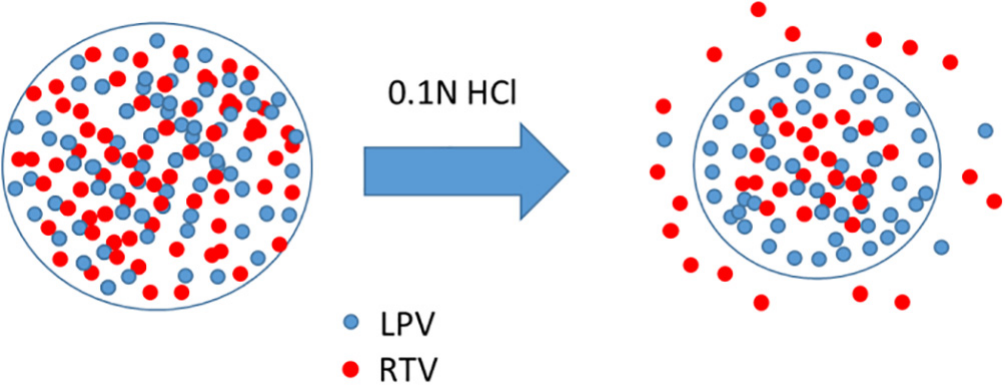
Proposed mechanism for retardation of RTV dissolution from co-amorphous system with LPV. Dissolution of RTV from the surface of the particles in acidic media leaves behind a LPV-rich surface which acts as a barrier for the remaining RTV to dissolve.

When the drug release profiles of the co-granulated sample and Aluvia^TM^ are compared in acidic media, it can be seen that the release of RTV is lower from the granules relative to the commercial tablets. It is desirable that the release behavior of the drugs prepared using wet granulation matches that of the innovator product, therefore it is of interest to understand potential causes for the differences. Most likely differences in drug:polymer ratios play a part in these observations, combined with the solubility suppression phenomenon in the co-amorphous mixtures. Drug release from an ASD typically falls within one of two regimens namely, drug-controlled or polymer-controlled. When the polymer is hydrophilic (such as PVPVA), polymer-controlled release will result in rapid drug dissolution. On the other hand, in a drug-controlled regime, slow drug dissolution would result for a hydrophobic drug. Whether the dissolution is drug-controlled or polymer-controlled depends on the drug-polymer ratio. The drug dissolution behavior of Aluvia^TM^ appears to be polymer-controlled since at pH 6.8, a milky solution was formed consistent with drug dissolution to above the amorphous solubility of each component, followed by liquid liquid phase separation and nanodroplet formation. Since nanodroplet formation during dissolution of ASDs is normally seen in the case of low drug loading (Purohit and Taylor, 2017), and specifically for RTV-PVPVA ASDs it has been shown to occur only for dispersions with a drug loading less than 25% (Indulkar et al., 2019), it is likely that the total drug loading in the marketed formulation is in this range or lower. In the wet granulated powders prepared herein, a higher drug loading dispersion was prepared (50%) to try and keep the final tablet weight at a reasonable value. In this regimen, we expect the drug release to be drug-controlled rather than polymer-controlled, based on previous observations with RTV and PVPVA dispersions (Indulkar et al., 2019). If only amorphous RTV was present in the ASD, then the drug release would have been fast and complete, since the drug ionizes upon contact with the medium and has a high driving force for dissolution. However, when present in combination with LPV, we expect the polymer to initially release faster than the drugs, leading to drug enrichment (Indulkar et al., 2019). Further, RTV will be preferentially released relative to LPV due to its higher solubility, thus the surface will become enriched with LPV after the initial release of RTV. At this point, the release will be mainly controlled by LPV, which is less soluble in acidic media since it does not ionize. This pattern of events is supported by evaluation of dispersions with high proportions of RTV, where a greater extent of RTV release is observed (Fig. 7). Further, a simple mass balance consideration supports the contention that LPV becomes enriched on the surface of the co-amorphous system after partial dissolution in acidic media. The remaining RTV now has to diffuse through a barrier layer that is LPV-rich, hindering complete dissolution. This proposed mechanism is shown in Fig. 11.

Since the presence of LPV intimately mixed with RTV resulted in retarded RTV release, even under favorable release conditions, i.e. in an acidic medium, the best approach appears to be of granulating the two drug separately and then either adding disintegrant and mixing prior to compaction or to make bi-layered tablets to physically separate the drugs. This approach led to much improved dissolution profiles, as shown in Fig. 8. Clearly this approach enables each drug to dissolve independently and better exploits the ionization of RTV in the acidic medium.

## 6 Conclusions

We have demonstrated proof-of-concept for the preparation of amorphous solid dispersions using a simple wet granulation approach. By dissolving drug(s) and polymer in an organic solvent, and adding to an excipient blend, compressible granules can be readily formed. No drug crystallinity existed after preparation, and the granules had good physical stability under accelerated storage conditions. This approach appears best suited for low or moderately low dose drugs, since the excipient burden required for this manufacturing method is relatively high. When preparing a co-amorphous formulation of ritonavir (minor component) and lopinavir (major component) using this approach, we found that the presence of lopinavir in the co-amorphous system depressed the release of ritonavir. This issue could be largely resolved by preparing individual ASD granules of each drugs, followed by mixing and compaction. Clearly the phase behavior of fixed dose combinations of amorphous formulations is highly complex and needs to be considered when designing the formulation.

## Declaration of Competing Interest

The authors declare that they have no known competing financial interests or personal relationships that could have appeared to influence the work reported in this paper.
