# Supplementary material for: Amorphous solid dispersion formation via solvent granulation – A case study with ritonavir and lopinavir
Source: Int J Pharm X. 2019 Nov 12;1:100035. doi: 10.1016/j.ijpx.2019.100035 (PMC6880113; doi:10.1016/j.ijpx.2019.100035)
Supplement: Supplementary data 1 [file mmc1.doc]

Supplemental Information

Amorphous solid dispersion formation via solvent granulation – a case study with ritonavir and lopinavir.

Niraj S. Trasi, Sonal Bhujbal, Qi Tony Zhou, Lynne S. Taylor

**Table S1. Summary of Compositions Evaluated**.

| **LPV (mg)** | **RTV (mg)** | **PVPVA (mg)** | **Surfactant (mg)** | **Lactose:MCC (mg)** | **Scale** |
| --- | --- | --- | --- | --- | --- |
| 200 | 0 | 200 | - | 1000 | Small |
| 0 | 50 | 50 | - | 400 | Small |
| 200 | 50 | 250 | - | 1500 | Small |
| 200 | 50 | 250 | - | 1500 | Bench-top |
| 125 | 125 | 250 | - | 1500 | Small |
| 50 | 200 | 250 | - | 1500 | Small |
| 200 | 50 | 1000 | - | 3000 | Small |
| 200 | 50 | 250 | 100mg Span | 1400 | Small |
| 200 | 50 | 250 | 2mg Tween | 1500 | Small |


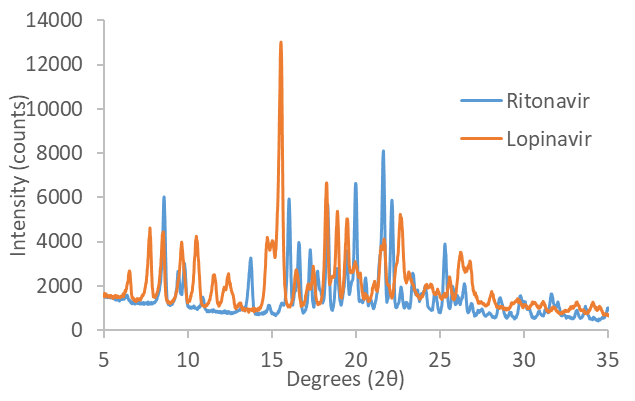


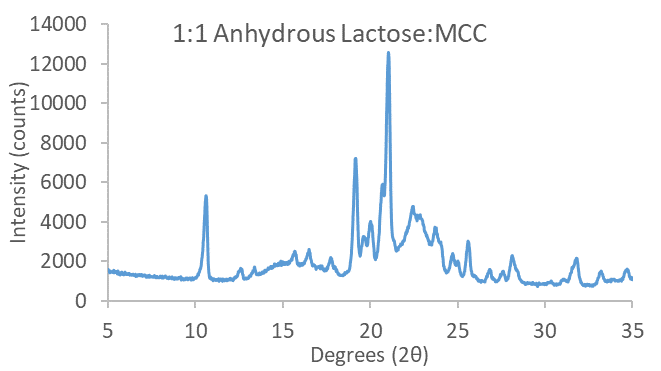

Figure S1. X-ray powder diffractograms of crystalline ritonavir and lopinavir (top) and a 1:1 mixture of anhydrous lactose and MCC (bottom).


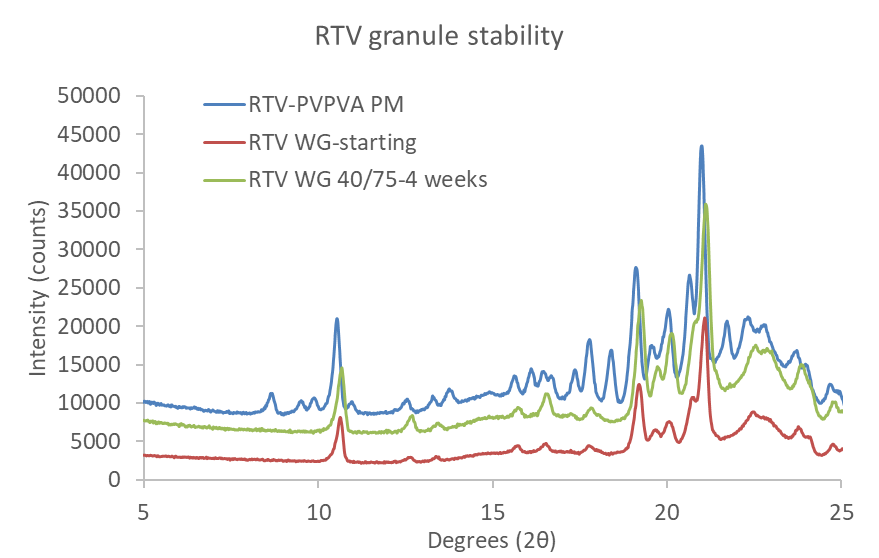


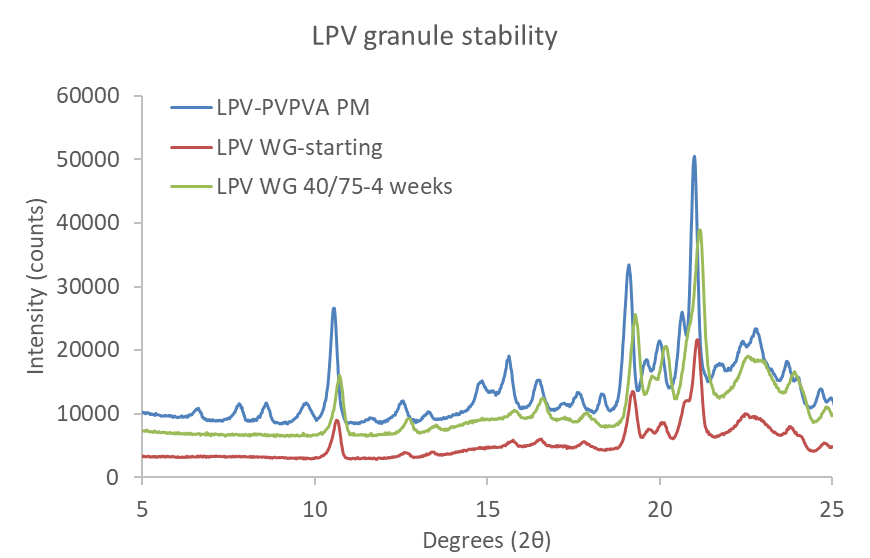


Figure S2. XRPD patterns of individually granulated drugs before and after storages at 40°C and 75% RH for 4 weeks showing that no crystallization occurred. The top panel shows ritonavir granules, while the bottom panel shows lopinavir granules. The drug loading was 50 wt. %.


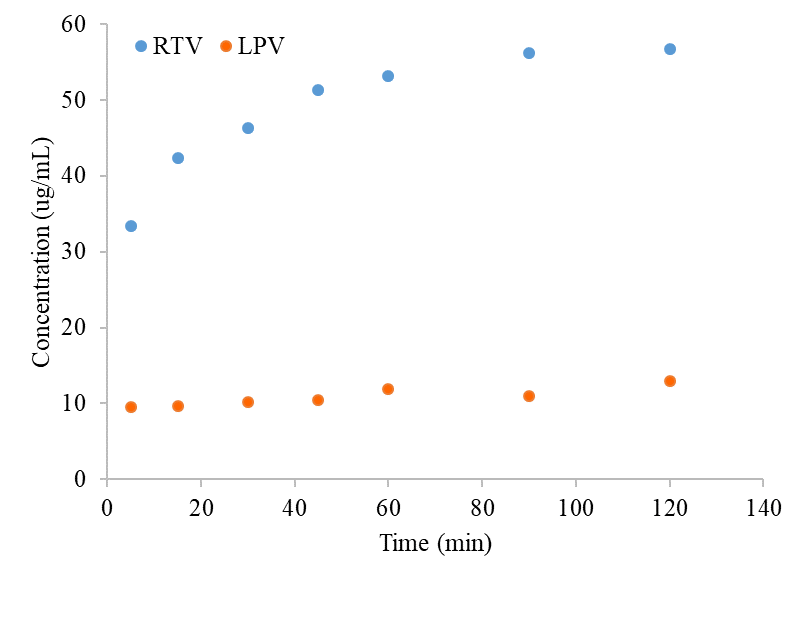


Figure S3. Dissolution of scaled up LPV-RTV granules in 100 mL 0.1N HCl at 37°C.


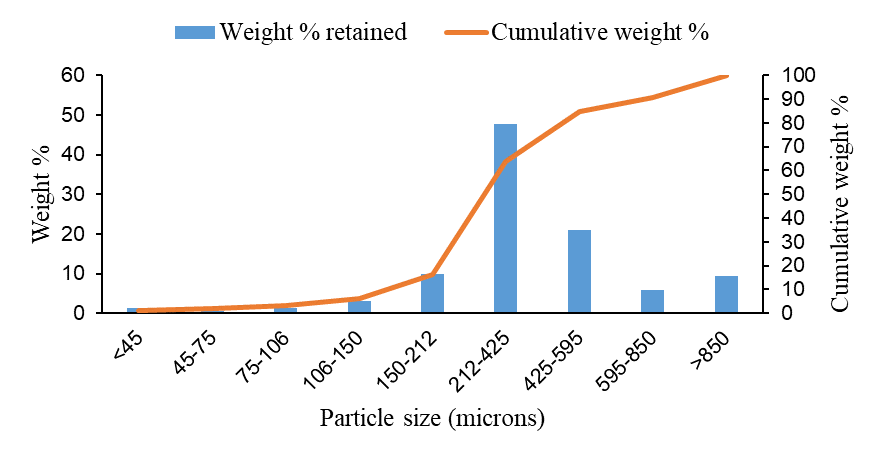


Figure S4. Percent weight of granules retained, and the cumulative particle size distribution as measured by sieve analysis
